# Outcomes following medical termination versus prolonged pregnancy in women with severe preeclampsia before 26 weeks

**DOI:** 10.1371/journal.pone.0246392

**Published:** 2021-02-03

**Authors:** Mariana A. Carvalho, Lina Bejjani, Rossana P. V. Francisco, Elizabeth G. Patino, Alexandre Vivanti, Fernanda S. Batista, Marcelo Zugaib, Frédéric J. Mercier, Lisandra S. Bernardes, Alexandra Benachi

**Affiliations:** 1 Faculdade de Medicina FMUSP, Universidade de Sao Paulo, Sao Paulo, Brasil; 2 Obstetrics and Gynecology Department, Hôpital Antoine-Béclère, Assistance Publique-Hôpitaux de Paris (AP-HP), Clamart, France; 3 Université Paris Saclay, Kremlin-Bicêtre, France; 4 Department of Anesthesia, Hôpital Antoine-Béclère, Assistance Publique-Hôpitaux de Paris (AP-HP), Clamart, France; Ospedale dei Bambini Vittore Buzzi, ITALY

## Abstract

**Objective:**

To compare maternal complications and describe neonatal outcomes in women with severe preeclampsia at ≤ 26^+0^ weeks in two countries with different management policies: expectant management (Brazil) versus termination of pregnancy (France).

**Methods:**

We conducted a retrospective comparative study by reviewing the medical records of women with severe preeclampsia at ≤ 26^+0^ weeks, from January 2010 to June 2018, in two centers: Hospital das Clínicas da Faculdade de Medicina, in Sao Paulo, Brazil (where medical abortion is forbidden in this indication) and Hôpital Antoine-Béclère, Clamart, France (where medical termination is accepted). We collected information on maternal characteristics, laboratory tests, maternal complications and fetal and newborn characteristics. We used Student’s t-test and the Mann-Whitney U nonparametric test to compare quantitative variables, and Chi-square test or Fisher's exact test to evaluate the associations between the qualitative variables.

**Results:**

There was no between-group difference in maternal complications during hospitalization (p = 0.846). In Brazil, the rate of cesarean section was 66.7%, and 20% of patients had vertical incision. The rate of spontaneous fetal death was 35.6% and among the live-born infants 26.6% were discharged from hospital. In France, one patient had a cesarean section with vertical incision.

**Conclusion:**

When comparing termination of pregnancy to expectant management in severe preeclampsia before 26 weeks, maternal complications were equivalent but maternal reproductive future might have been compromised in 20% of cases due to a higher risk of uterine rupture in subsequent pregnancies for patients having classic cesarean (vertical incision). 26.6% of children survived the neonatal period when pregnancy was pursued, however we lack information on their long-term follow-up.

## Introduction

Preeclampsia is a pregnancy-specific hypertensive disorder that can rapidly progress to serious complications with maternal and/or fetal morbidity and mortality. In 2011, hypertensive disorders in pregnancy accounted for 25% of maternal deaths in Latin America [1]. In Brazil, the reported rate of preeclampsia is about 1.5% but is probably underestimated, on the one hand because of lack of official data and on the other hand because of regional differences with rates varying from 0.2% to as high as 8% in some regions [2]. In France the estimated rate of preeclampsia was about 2% in 2016, with 12.2% of cases diagnosed before 28 weeks of pregnancy [3].

The only definitive treatment of preeclampsia is delivery, but whether delivery should be immediate or delayed depends on a set of arguments including gestational age, the severity of maternal disease and fetal well-being. The recommended management is mainly based on expert opinion and few studies have evaluated management in early-onset severe preeclampsia [4–9]. To date, no study has compared maternal complications with expectant management versus termination in women diagnosed with severe preeclampsia at less than or equal to 26^+0^ weeks. In France, in severe preeclampsia between 24 and 26 weeks of gestation, expectant management or termination of pregnancy (TOP) can be discussed in the case of severe maternal disease, especially if associated with fetal growth restriction (FGR) [5, 10]. However, in severe preeclampsia before 24 weeks of gestation, counseling of patients is in favor of TOP given the high risk of maternal complications and the small chance of perinatal survival [11]. It should be noted that in France TOP is authorized by law and may be performed at any stage of gestation if there is a strong possibility that the unborn child is suffering from a serious incurable condition or if the mother has a life-threatening condition [12]. In Brazil, as in many other countries where abortion is illegal in all weeks of gestation, except in cases of rape, expectant management is the standard management for severe preeclampsia remote from term, even in the pre-viable period (less than 24^+0^ weeks).

In an attempt to compare maternal complications and describe neonatal outcomes for those two different management policies, we decided to conduct a retrospective study comparing two groups of pregnant women diagnosed with severe preeclampsia ≤ 26^+0^ weeks of gestation in two centers: Hospital das Clínicas da Faculdade de Medicina Hospital da Universidade de Sao Paulo (HCFMUSP) in Brazil and Hôpital Antoine-Béclère (HAB) in France.

## Methods

We retrospectively reviewed the medical records of pregnant women with severe or severe superimposed preeclampsia ≤ 26+0 weeks, between January 2010 and June 2018, in two centers: HCFMUSP in Brazil and HAB in France. This study was approved by both centers’ institutional review boards (CEROG N° 2019-OBST-0801; CAAE 19179913.4.0000.0068). Regarding patient records, the data was obtained through access to electronic and physical medical records and were anonymized after collection. Ethics committees waived the requirement for informed consent because the research was retrospective and did not interfere with the care received by the patient.

Inclusion criteria were singleton pregnancies, gestational age ≤ 26^+0^ weeks at admission, fetus alive at admission, absence of fetal malformations and at least one criterion of severe preeclampsia based on the 2013 task force report “hypertension in pregnancy “, since these patients were managed before the 2019 guidelines. Exclusion criteria were pregnant women in the HAB group who chose not to undergo pregnancy termination. In both hospitals, the check-up was performed by obstetricians every 2–4 weeks. In Brazil few patients were transferred to the hospital after the diagnosis in primary care service so in these cases a delay in diagnosis and treatment could have happened.

Diagnosis of severe preeclampsia was established when at least 1 severity criteria was present: blood pressure (BP) ≥160 mm Hg systolic and/or ≥110 mm Hg diastolic on 2 occasions at least 6 h apart while patient is on bed rest; proteinuria ≥5 g in 24-h urine specimen; oliguria <500 mL in 24 h or creatinine ≥ 1.1 mg/dL; cerebral symptoms (eclampsia, stroke) or visual symptoms; pulmonary edema or cyanosis; epigastric or right upper quadrant pain; HELLP syndrome (aspartate aminotransferase and/or alanine aminotransferase ≥ 70 mg/dL or twice normal; platelets < 100,000/mm^3^, total bilirubin ≥ 1.2 mg/L or lactic dehydrogenase ≥ 600 mg/dL); impaired liver function; thrombocytopenia [13]. Gestational age was determined by the last menstrual period and confirmed by the earliest ultrasound measurements. Gestational age was calculated using crown-rump length, if there was a disagreement of ≥7 days between the last menstrual period and crown-rump length measurements before 14 weeks of pregnancy [14], and by fetal biometry when the difference in age based on the last menstrual period was ≥10 days between 14 and 16 weeks of pregnancy [14].

Admission fetal evaluation included an ultrasound scan to determine viability, fetal weight estimation using Hadlock’s reference curve [14] and Doppler velocimetry of the umbilical and middle cerebral arteries to evaluate placental and fetal vascular resistances. In HCFMUSP the ductus venosus Doppler was also performed in cases with high umbilical resistance.

### Group HCFMUSP-Brazil

Patients were hospitalized and closely monitored by the on-call team. BP monitoring and blood analysis were performed every 4 to 6 hours, 24-h urine collection was started, and urine input-output was regularly monitored as was the neurological state of the patient. Intravenous magnesium sulfate was administered in the case of imminent eclampsia and during progression of labor. During maternal hospitalization, initial bolus injections of hydralazine was used to decrease BP in cases of symptomatic systolic BP > = 160 mm Hg or diastolic BP > = 110 mm Hg. Oral antihypertensive medications were then used to control BP and the drugs more frequently used were methyldopa, pindolol and amlodipine. Fetal well-being was assessed by regular biophysical profile and fetal Doppler. Delivery was indicated on identification of maternal worsening, defined as failure to control BP (diastolic BP >110 mm Hg despite combined oral or intravenous antihypertensive treatment on maximum dose), continuous worsening of laboratory findings with associated HELLP, or the development of major maternal complications (cerebral or hepatic hematoma, pulmonary edema, eclampsia, abruptio placenta and disseminated intravascular coagulation). The indications for delivery because of fetal worsening were: the presence of non-reassuring decelerations in continuous antepartum fetal heart rate monitoring, significant changes in Doppler evaluation such as reverse end diastolic flow in the umbilical artery associated with ductus venosus pulsatility index above 1.5, or pulsatile umbilical venous flow and reversed a-wave in ductus venosus. Another indication for delivery was fetal death.

### Group HAB-France

Upon arrival, patients were evaluated with continuous BP monitoring, blood and urine workup. On-call obstetrical and anesthesiology teams performed clinical evaluation. First-line antihypertensive drugs used for severe preeclampsia were intravenous nicardipine hydrochloride or labetalol or their combination; when necessary urapidil was used as a third-line therapy. According to French recommendations the objective of treatment was to maintain systolic BP ≤150 mmHg and diastolic BP ≤ 100 mmHg. Intravenous magnesium sulfate was administered only in the case of maternal neurological symptoms or for fetal central nervous system protection in the case of considered delivery with active neonatal support.

The nurses, midwives, obstetrical and anesthesiology teams closely monitored patients in labor rooms. BP monitoring and blood analysis were performed every 4 to 6 hours, 24-h urine collection was started, and urine input-output was regularly monitored as was the neurological state of the patient. The senior on-call obstetrician performed ultrasound evaluation of fetal growth, and patients were counseled by the obstetric and neonatology teams. The management was determined depending on the condition of both mother and fetus. If a delivery with active neonatal support was considered, fetal monitoring by CTG was initiated.

In the case of a severe preeclampsia with onset before 26 weeks of gestation, interventionist care was discussed with the patient, generally within the first 48 hours, especially in the case of unstable maternal or fetal condition. Until 2016, active neonatal resuscitation was not generally performed before 25 weeks of gestation and TOP could be discussed given the increased risk of maternal morbidity in the case of prolongation of pregnancy without any fetal benefit. Delivery by cesarean section could be offered in pregnancies between 25 and 26 weeks with an estimated fetal weight ≥ 600g and with normal fetal parameters (umbilical cord Doppler, cardiotocography), after a full course of corticosteroids and a course of magnesium sulfate. Counseling of the parents emphasizes on the one hand the high risk of neonatal morbidity and mortality given the extreme prematurity and the lack of accuracy in fetal weight estimation (15% error margin), and on the other hand the obstetrical morbidity if a classic cesarean section (vertical incision in the uterus) is performed, which carries a significant risk of uterine rupture and placenta abnormalities such as placenta accreta and percreta in subsequent pregnancies, instead of low transverse C-section, which is associated with lower obstetrical morbidity. It is seldom the case since severe preeclampsia is often associated with severe fetal growth restriction and termination is allowed after a multidisciplinary discussion in this situation of conflicting maternal and fetal interests with a compromised prognosis in severely growth-restricted fetuses. Starting 2016, the weight threshold was lowered to 500g and the limit of viability to 24–25 weeks.

When a TOP is decided, induction of fetal demise is performed via a sonographically guided umbilical cord administration of 5 μg of sufentanil followed by injection of 20 mL of lidocaine 1% under epidural anesthesia or local anesthesia depending on platelet count and its time course. This procedure helps stabilize maternal disease before induction of labor. Patients are then administered 200 mg of mifepristone and osmotic cervical dilators are inserted in the cervix and left in place for 4 to 6 hours, depending on the stability of the maternal condition. Cervical dilators allow cervical ripening and rupture of membranes prior to induction of labor with misoprostol usually performed under epidural anesthesia unless contraindicated. After delivery, patients are monitored in the recovery room or the ICU or in post-partum rooms if they are considered stable. The placenta is submitted to pathological evaluation and fetal autopsy is systematically discussed with parents.

The maternal characteristics collected were age; parity; previous miscarriages; comorbidities (hypertensive disease, diabetes mellitus, immunological diseases and renal insufficiency); gestational age at onset; BP at onset; laboratory tests at onset; days of hospitalization; days gained until birth in live fetus; use of corticosteroids (betamethasone 12 mg/d for 2 days); type of anesthesia, delivery and cesarean section (low transverse cesarean or classic cesarean); indication for delivery in the expectant group (maternal worsening, fetal worsening or both); intraoperative or postoperative complications; maternal complications (HELLP or partial HELLP syndrome, abruptio placenta, acute renal failure, subcapsular liver hematoma, pulmonary edema, ascites, coagulopathy, cerebrovascular accident, eclampsia and maternal death occurring before or after fetal death or delivery); changes in laboratory and clinical parameters after fetal death or delivery; decreased antihypertensive therapy (dose reduction or intravenous to oral switch) within 48 h after fetal death or delivery and improvement in laboratory parameters (creatinine levels, aspartate aminotransferase, alanine aminotransferase or platelets) in two consecutive exams within 48 h after the fetal death or delivery. The fetal and neonatal characteristics evaluated were estimated fetal weight at admission; end-diastolic flow at umbilical artery; gestational age of spontaneous intrauterine demise, termination, and live birth; birth weight of intrauterine death fetuses and neonates; days gained until birth in liveborn fetuses; newborn sex; neonatal outcome (death, perinatal survival); Apgar scores; hospitalization days, neonatal complications (bronchopulmonary dysplasia, necrotizing enterocolitis, intracranial hemorrhage and sepsis) and age at hospital discharge.

Quantitative variables are expressed as mean, standard deviation (SD), median, minimum, and maximum and the qualitative variables are presented as *n* and a percentage. To verify the normal distribution of the data we used the Shapiro-Wilk and Kolmogorov-Smirnov tests. To compare quantitative variables between the two independent groups, Student’s t-test was used when the variables had a normal distribution; otherwise, the Mann-Whitney U nonparametric test was used. To evaluate the associations between the qualitative variables, a *χ*^2^ test (Chi-square test) or Fisher's exact test was used as appropriate. A p value < 0.05 was considered significant. The statistical analyses were performed using SPSS 20.0.

## Results

A total of 80 patients hospitalized during the study period were initially defined eligible, of whom 4 in HAB were excluded because they opted for conservative management. The final sample consisted of 45 patients from group HCFMUSP and 31 patients from group HAB.

Maternal characteristics are shown in Table 1. The only significant clinical differences between the groups were parity, the duration of hospitalization, the cesarean section rate and the use of general anesthesia. In group HCFMUSP the rate of cesarean section was high (66.7%) and 20% of patients had classic cesarean section (vertical incision), but there was no relevant complication, neither blood transfusion nor postoperative reintervention; only one patient had a scar hematoma but it did not require surgery. In group HAB, only one patient had a classic cesarean section because of large previa myoma; there were no postoperative reintervention, and 3 patients received platelets and/or red blood cells during the induction procedure.

**Table 1:** Maternal characteristics.

Maternal Characteristics	HCFMUSP		HAB		
	N = 45		N = 31		
N = 76	Mean ± SD / n (%)	Median (Minimum -Maximum)	Mean ± SD / n (%)	Median (Minimum—Maximum)	p-value
**Maternal age (yr)**	31.9 **±** 6.9	32 (18–46)	32.4 **±** 7.2	31 (21–53)	0.752 (T)
**Parity**					**0.021** (x^2^)
Nulliparous	25 (55.6)		25 (80.6)		
Multiparous	20 (44.4)		6 (19.4)		
**Previous abortion**	13 (28.9)		10 (32.3)		0.754 (x^2^)
**GA at onset (wk)**	24.0 ± 1.2	24.0 (21.4–25.7)	23.8 ± 1.2	24.1 (21.0–25.6)	0.561 (MW)
**SBP at onset**	169.7 ± 14.9	170 (140–200)	169.5 ± 17.2	170 (145–220)	0.855 (MW)
**DBP at onset**	106.5 ± 11.1	110 (80–140)	102.3 ± 9.4	100 (86–120)	0.059 (MW)
**Hospitalization days**	13.2 ± 7.3	11 (2–33)	9.0 ± 3.0	8.00 (5–18)	**0.022** (MW)
**Antenatal steroids**	19 (50)		11 (35.5)		0.225 (x^2^)
Missing	7		0		
Complete course	15 (78.9)		2 (18.2)		0.126 (F)
**Magnesium sulfate**	6 (14)		6 (19.4)		0.536 (x^2^)
**Anesthesia**	43 (95.6)		31 (100)		0.511 (F)
Spinal/Epidural	35 (81.4)		30 (96.8)		-
General	8 (18.6)		0		**0.018** (F)
PCA with sufentanil	0		1 (3.2)		-
**Indication for delivery**					
Maternal	14 (31.1)		-	-	-
Fetal	17 (37.8)		-	-	-
Fetal death	14 (18.4)		-	-	-
**Birth type**					
Classic C-section (vertical)	9 (20)		1 (3.2)	-	-
Low transverse C-section	21 (46.7)		-	-	-
Vaginal	15 (33.3)		30 (96.8)	-	**< 0.001** (F)

HCFMUSP: Hospital das Clínicas da Faculdade de Medicina da Universidade de Sao Paulo; HAB: Hôpital Antoine-Béclère SD: standard deviation; yr: years; GA: gestational age; wks: weeks; SBP: Systolic blood pressure; DBP: Diastolic blood pressure; Student’s t- test (T); Mann-Whitney test (MW); Chi-square test (x^2^).

Table 2 shows that the groups were similar regarding laboratory abnormalities at admission, but they were different in terms of maternal comorbidities because group HCFMUSP had a higher prevalence of previous chronic hypertension (p < 0.001). There was no difference between the groups regarding the rate of maternal complications during the hospitalization period (68.9% in group HCFMUSP and 71% in group HAB, p = 0.846) and there were no cases of cerebrovascular accident, eclampsia and maternal death. Furthermore, there was no significant difference between the groups regarding improvement in laboratory parameters within 48 h of the event considered as fetal death or delivery, but there was a significant difference regarding the step-down antihypertensive therapy within 48 h after the event (24% in group HCFMUSP and 80% in group HAB, p < 0.001) (Table 3). In group HCFMUSP, 13 patients had normal laboratory test results before delivery or spontaneous fetal death.

**Table 2:** Maternal comorbidities and exams at admission.

Maternal comorbidities and exams on admission	HCFMUSP	HAB	
	N = 45	N = 31	
N = 76	n (%)	n (%)	p value
**Maternal comorbidities**	26 (57.8)	3 (9.7)	**< 0.001 (F)**
Chronic hypertension	22 (48.9)	2 (6.5)	**< 0.001 (F)**
Preexistent diabetes	4 (8.9)	0	0.141 (F)
Immunological disease	5 (11.1)	0	0.075 (F)
Renal insufficiency	2 (4.4)	0	0.511 (F)
**Exams at admission**			
AST > 70	8 (17.8)	6 (19.4)	0.862 (x^2^)
ALT > 70	11 (24.4)	8 (25.8)	0.893 (x^2^)
Creatinine ≥ 1.2 mg/dl	4 (10)	1 (3.2)	0.378 (F)
Platelets < 100 000	9 (20.5)	4 (12.9)	0.539 (F)

HCFMUSP: Hospital das Clínicas da Faculdade de Medicina da Universidade de Sao Paulo; HAB: Hôpital Antoine-Béclère; AST: aspartate aminotransferase; ALT: alanine aminotransferase; Chi-square test (x^2^); Fisher’s exact test (F).

**Table 3:** Maternal complications during hospitalization.

Maternal complications	HCFMUSP	HAB	
	N = 45	N = 31	
N = 76	n (%)	n (%)	p value
Complications overall	31 (68.9)	22 (71)	0.846 (x^2^)
HELLP	23 (51.1)	17 (54.8)	0.749 (x^2^)
Abruptio placenta	2 (4.4)	0 (0.0)	0.511 (F)
Acute renal failure	13 (28.9)	9 (29)	0.989 (x^2^)
Subscapular liver hematoma	1 (2.2)	0 (0.0)	1.000 (F)
Pulmonary edema	1 (2.2)	3 (9.7)	0.298 (F)
Ascites	3 (6.7)	2 (6.5)	1.000 (F)
Coagulopathy	0 (0.0)	1 (3.2)	0.408 (F)
Timing of complications:			
Before event *	28 (90.3)	15 (71.4)	0.133 (F)
After event *	3 (9.7)	6 (28.6)	

HCFMUSP: Hospital das Clínicas da Faculdade de Medicina da Universidade de Sao Paulo; HAB: Hôpital Antoine-Béclère; Event was considered as spontaneous fetal death, pregnancy termination or birth (the first event); Chi-square test (x^2^); Fisher’s exact test (F).

Table 4 describes fetal and newborn characteristics in the expectant group (group HCFMUSP, n = 45). Spontaneous fetal death occurred in 16 cases (35.6%). The mean gestational age at delivery of live-born was 26.5 weeks. Among the 29 live-born infants, the perinatal mortality rate was 55.2% (n = 16). Median hospitalization time of the neonates who died was 6.5 days (range 1 to 53). Of the remaining 13 cases, 1 case was lost to follow-up after being transferred, and 12 patients were discharged from hospital. Their characteristics are detailed in Table 5. The most common clinical complications were bronchopulmonary dysplasia and sepsis. Two newborns were discharged with home oxygen therapy.

**Table 4:** Fetal and newborn characteristics.

Fetal/Newborn Characteristics	HCFMUSP		HAB		
	N = 45		N = 31		
N = 76	Mean ± SD / n (%)	Median (Minimum–Maximum)	Mean ± SD / n (%)	Median (Minimum–Maximum)	p value
Estimated Fetal weight at admission (g)	500.6 **±** 176.7	507 (186–940)	451.2 **±** 110.2	440 (247–641)	0.145 (T)
Percentile < 3 at admission	31 (72.1)	-	22 (71)	-	0.916 (x^2^)
Absent end-diastolic flow	17 (42.5)	-	8 (26.7)	-	0.168 (x^2^)
Missing	5		1		
Fetal outcome:					
Spontaneous fetal death/termination	16 (35.6)	-	31 (100)	-	-
Newborn death	16 (35.6)	-	-	-	-
Newborn discharge	12 (26.7)	-	-	-	-
Missing	1	-	-	-	-
Birth weight of live-born (g)	647.4 ±159.3	620 (340–970)	-	-	-
GA of fetus live-born	26.5 ± 1.3	26.7 (22.9–29)	-	-	-
Birth weight of intrauterine death fetus (g)	356.1 **±** 108.9 /	378 (170–550)	435.8 ± 102.3 /	450 (240–620)	**0.016 (MW)**
GA of spontaneous fetal death/termination (wk)	23.9 ± 1.2	23.9 (21.9–26.1)	24.1 ± 1.2	24.3 (21.1–25.7)	-
Newborn sex					
Male	17 (42.5%)	-	11 (35.5)	-	0.548 (x^2^)
Female	23 (57.5%)		20 (64.5)		
Missing	5		-		
Days gained until birth (only live fetuses)	10.8 ± 7.4	9 (0–27)	-	-	-
Survival days of neonates that died	10.7 ± 13.7	6.5 (1–53)	-	-	-

HCFMUSP: Hospital das Clínicas da Faculdade de Medicina da Universidade de Sao Paulo; HAB: Hôpital Antoine-Béclère; SD: standard deviation; g: grams; wk: weeks; d: days; GA: gestational age; Student’s t-test (T); Mann-Whitney test (MW); Chi-square test (x^2^).

**Table 5:** Characteristics of newborns discharged from hospital.

Newborn Characteristics	HCFMUSP	
N = 12	Mean ± SD / N (%)	Median (Minimum–Maximum)
Hospitalization (d)	83.1 ± 14.8	82.5 (57–106)
Newborn weight	725.4 **±** 138.0	715 (490–970)
GA at birth	27.0 **±** 1.6	27.0 (22.9–29)
1-minute Apgar score	4.2 **±** 2.4	4 (1–8)
5-minute Apgar score	7.7 **±** 1.5	8.5 (6–9)
10-minute Apgar score	9.2 **±** 0.6	9 (8–10)
Sex		
Male	4 (33.3)	
Female	8 (66.7)	
Bronchopulmonary dysplasia * (n = 11)	9 (81.8)	
O_2_ home therapy *	2 (18.2)	
Necrotizing enterocolitis *	0	
Cerebral hemorrhage (III/IV) *	1 (9)	
Sepsis *	9 (81.8)	

HCFMUSP: Hospital das Clínicas da Faculdade de Medicina da Universidade de Sao Paulo; SD: standard deviation; d: days; GA: gestational age

* we used n = 11, because 1 case was not considered as we had no access to data on neonatal complications during hospitalization.

We also evaluated the fetal and neonatal outcomes by gestational age of preeclampsia onset in Group HCFMUSP. 87.5% of intrauterine death cases occurred when preeclampsia started before 24^+1^ weeks and the perinatal survival rate ranged between 18% when onset occurred before 23^+1^ weeks, and 39% when onset occurred after 25^+0^ weeks. The number of days gained until delivery was higher for more advanced gestational age of preeclampsia onset (mean of 5 days before 23^+1^ weeks and 13 days after 25^+0^ weeks).

## Discussion

The most important result of our study is that the rate of maternal complications was similar, although the approaches were opposite between both centers (expectant versus TOP), in other words, even when the pregnancy was prolonged, on average by 11 days (fetus alive), there was no difference of short-term maternal complications, with no cases of maternal death or eclampsia. However, in the expectant group (HCFMUSP), 20% of patients had a classic cesarean section, which carries a risk of 4 to 9% of uterine rupture in subsequent pregnancies [15, 16]. We also observed that although complication rates were not different in the two populations, the duration of hospital stay was significantly shorter for patients managed with TOP compared to expectantly managed patients (13.2 ±7.3 v/s 9.0 ± 3.0), with a bigger proportion of clinical recovery within the first 48 hours of termination. Regarding perinatal outcomes, the survival rates were low with expectant management with a mean survival rate of 26.6%, and the complication rates were also high, ranging from 9% for cerebral hemorrhage to 81.9% for sepsis and bronchopulmonary dysplasia.

Few studies have evaluated the management of early-onset severe preeclampsia, and our results seem relatively consistent with previous conclusions [4–9]. The most recent paper by Van Oostwaard MF et al. [4] described the maternal and neonatal outcomes of pregnancies with severe early-onset preeclampsia between 22 and 26 weeks in Dutch tertiary perinatal care centers between 2008 and 2014 in a population of 133 women and 140 children. Delivery occurred at a median gestational age of 25 weeks, mostly for maternal indications and after a median prolongation of 5 days. Maternal complications were seen in 54% of cases, and neonatal survival was 19% among the 50 newborns who received active neonatal support. The rate of neonatal complications among the survivors was 85%.

In this setting prolongation of pregnancy might be discussed with parents and, considering the downside of prolongation, parental counseling should balance the risk for maternal complications with the limited neonatal survival and/or extreme prematurity, and its immediate and future morbidity. Babies who are both small for their age and premature suffer additional complications, with an increased risk of perinatal morbidity and mortality and health implications across their lifespan [17, 18]. Patients should be informed of the limited accuracy of ultrasound in the estimation of fetal weight as there is an inherent systematic error that can significantly impact the detection of FGR, which adds an extra layer of uncertainty to the decisional process [19].

The discussion with parents should also consider the current legislation in the country the patient is in, each center’s experience and results as well as familial values in order to provide adequate care [20]. Guillen et al. recently evaluated the use of a decision-making tool to inform parents with a high risk of extreme prematurity in order to investigate values and to ensure an informed parental decision concerning neonatal artificial life support. The authors observed that the use of this tool was related to greater understanding of complex information [21]. This kind of tool could be studied in the population with early-onset severe preeclampsia in order to explain maternal risks and perinatal survival and complications for joint parental and medical decision-making.

TOP is still a controversial issue, especially in countries like Brazil where religious and cultural beliefs prevail and where law forbids such a procedure except under special circumstances like rape or conditions that are an imminent danger to maternal life. Considering the poor survival rates and the maternal burden, government and experts should consider offering active management and induction of labor with exclusive comfort care support for the neonate when onset occurs before 24 weeks of gestation or when there is severe FGR.

Major limitations to our study are its retrospective design and the small number of patients included. However, the number of patients evaluated included the total population followed up in two major referral centers over 9.5 years, making the study clinically relevant.

A further limitation is that the two groups were not comparable in terms of the antihypertensive drugs used, since all patients managed in HAB received intravenous antihypertensive therapy while oral antihypertensive drugs were exclusively used in HCFMUSP patients. A recent study by Easterling et al. [22] showed that oral antihypertensive drugs are effective in reducing BP and our data show that complications were similar in the two groups of patients, suggesting that both antihypertensive regimens might be used. Further studies should focus on comparing those regimens in reducing maternal complication rates and prolonging pregnancy in cases of severe pre-eclampsia before term. Another difference between management of cases between centers is the use of magnesium sulfate. Although the use of magnesium sulfate is indicated for neurological protection of premature fetuses only at HAB, the rate of patients that receive the drug in case of maternal neurological symptoms was similar between centers. A final limitation of our study is the lack of data on the long-term follow-up of the living infants and of the mothers. Further studies should focus on investigating the long-term neurological development of these infants and the long-term maternal complications [23].

The major strength of our study is to compare opposing management policies of early severe preeclampsia in two major centers, and despite being a retrospective study, the inclusion of patients was considered using the same diagnostic criteria for severe preeclampsia and also the patients’ clinical evaluation and laboratory tests on admission of both groups were similar.

In conclusion, when comparing termination of pregnancy to expectant management in severe preeclampsia before 26 weeks in our population, maternal complications were equivalent but maternal reproductive future might have been compromised in 20% of cases due to a higher risk of uterine rupture in subsequent pregnancies for patients having classic cesarean (vertical incision). Twenty-six percent of children survived neonatal period when pregnancy was pursued, but we lack information on long-term outcome. Given the high risk of maternal and the neonatal morbidity and mortality, large prospective studies are needed to improve the management of early-onset severe preeclampsia. Potential criteria defining a select group of patients who can benefit from a relatively safe pregnancy prolongation should be evaluated by future studies; the long-term neurological development of living infants and the long-term maternal complications must be investigated as well.
